# Impact of ATP-citrate lyase catalytic activity and serine 455 phosphorylation on histone acetylation and inflammatory responses in human monocytic THP-1 cells

**DOI:** 10.3389/fimmu.2022.906127

**Published:** 2022-11-10

**Authors:** Monica Dominguez, Verena Truemper, Ana Carolina Mota, Bernhard Brüne, Dmitry Namgaladze

**Affiliations:** ^1^ Institute of Biochemistry I, Faculty of Medicine, Goethe-University Frankfurt, Frankfurt, Germany; ^2^ Fraunhofer Institute for Translational Medicine and Pharmacology (ITMP), Frankfurt, Germany; ^3^ German Cancer Consortium (DKTK), Partner Site Frankfurt, Frankfurt, Germany; ^4^ Frankfurt Cancer Institute, Goethe-University Frankfurt, Frankfurt, Germany

**Keywords:** ATP-citrate lyase, histone acetylation, macrophages, metabolism, inflammation

## Abstract

ATP-citrate lyase (ACLY) is a key enzyme provoking metabolic and epigenetic gene regulation. Molecularly, these functions are exerted by the provision of acetyl-coenzyme A, which is then used as a substrate for *de novo* lipogenesis or as an acetyl-group donor in acetylation reactions. It has been demonstrated that ACLY activity can be positively regulated *via* phosphorylation at serine 455 by Akt and protein kinase A. Nonetheless, the impact of phosphorylation on ACLY function in human myeloid cells is poorly understood. In this study we reconstituted ACLY knockout human monocytic THP-1 cells with a wild type ACLY as well as catalytically inactive H760A, and phosphorylation-deficient S455A mutants. Using these cell lines, we determined the impact of ACLY activity and phosphorylation on histone acetylation and pro-inflammatory gene expression in response to lipopolysaccharide (LPS). Our results show that ACLY serine 455 phosphorylation does not influence the proper enzymatic function of ACLY, since both, wild type ACLY and phosphorylation-deficient mutant, exhibited increased cell growth and histone acetylation as compared to cells with a loss of ACLY activity. Transcriptome analysis revealed enhanced expression of pro-inflammatory and interferon response genes in ACLY knockout and H760A THP-1 cells under unstimulated or LPS-treated conditions. At the same time, S455A ACLY-expressing cells showed a phenotype very similar to wild type cells. Contrary to ACLY knockout, pharmacological inhibition of ACLY in THP-1 cells or in primary human macrophages does not enhance LPS-triggered pro-inflammatory gene expression. Our data thus suggest that ACLY retains functionality in the absence of Akt/PKA-mediated phosphorylation in human myeloid cells. Furthermore, loss of ACLY activity may elicit long-term adaptive mechanisms, increasing inflammatory responses.

## Introduction

Macrophages are highly plastic immune cells. They adapt to changes in their environment to serve diverse physiological functions from defense against pathogens to tissue repair (1, 2). Profound metabolic changes often accompany macrophage responses to environmental challenges, such as up-regulation of glycolysis during macrophage responses to infection. At the same time, multiple metabolites regulate immune responses (3). Metabolic reprogramming of macrophages has been described as a promising therapeutic target to combat inflammatory disorders (4, 5).

A key enzyme linking metabolism to control of gene expression is ATP-citrate lyase (ACLY). This homotetrameric enzyme, which is localized in the cytosol and nucleus, catalyzes the production of acetyl-coenzyme A (acetyl-CoA), cleaving citrate exported from mitochondria *via* the SLC25A1 transporter (6). Acetyl-CoA produced by ACLY is a central metabolite involved in epigenetic and transcriptional regulation through its use as a substrate for histone and protein acetylation (7, 8). Acetyl-CoA is also a substrate for *de novo* synthesis of fatty acids and sterols necessary for rapid cell growth, making ACLY an attractive target for anti-cancer therapies (9–11). ACLY is also suggested to play important roles in the innate immune system by its contribution to the epigenetic control of pro- and anti-inflammatory responses (6). However, contrasting findings are reported in the literature regarding the impact of ACLY on pro-inflammatory gene expression. Thus, in murine macrophages, pharmacological ACLY inhibition or ACLY silencing decreased histone acetylation and reduced pro-inflammatory gene expression after stimulation with lipopolysaccharide (LPS) (12, 13). In contrast, recent *in vivo* and *in vitro* studies of myeloid-specific ACLY deficiency reported increased pro-inflammatory gene expression in macrophages and atherosclerotic plaques (14, 15). While the effects of ACLY on murine macrophage pro-inflammatory or alternative polarization have recently been described both *in vitro* and *in vivo*, we are still uncertain of the effects ACLY exhibits in human macrophages.

ACLY is known to be phosphorylated at serine 455, influencing its enzymatic activity (16). Akt (17) and protein kinase A (PKA) (18) are major serine 455 kinases. Studies have demonstrated that in tumor cells, upon glucose deprivation Akt-dependent phosphorylation at serine 455 supports acetyl-CoA production and histone acetylation (19). Notably, in interleukin-4-stimulated murine bone marrow-derived macrophages (BMDMs), ACLY phosphorylation at serine 455 was induced in Akt-dependent manner (20). Additionally, pharmacological inhibition of ACLY or Akt attenuated histone H3 and H4 acetylation at promoters of Akt-sensitive target genes. These observations correlated with decreased mRNA expression of Akt-responsive M2 target genes (20). Similarly, Lauterbach et al. described Akt-dependent phosphorylation of serine 455 upon LPS stimulation, which was abolished upon inhibition of toll-like receptor signaling (12). Nonetheless, how ACLY phosphorylation at serine 455 is regulated in human myeloid cells and how this influences gene expression upon pro-inflammatory stimulation remains to be investigated.

Given the contradictory findings on the role of ACLY in macrophage polarization (6), we made use of the ACLY knockout human monocytic THP-1 cell line, described by us previously (21). By re-expressing wild type, catalytically inactive H760A, and phosphorylation-deficient S455A ACLY mutants, we showed that basal ACLY serine 455 phosphorylation does not influence enzymatic ACLY function in THP-1 cells. Moreover, attenuating ACLY in THP-1 cells showed a pro-inflammatory phenotype similar to that of knockout BMDMs (14), an effect not recapitulated by pharmacological inhibition of ACLY.

## Materials and methods

### Cell culture and treatment

Human peripheral blood mononuclear cells were isolated from commercially obtained buffy coats from anonymous donors (DRK Blutspendedienst Baden-Württemberg-Hessen, Institut für Transfusionsmedizin und Immunhämatologie, Frankfurt, Germany) using Ficoll (Biochrom, cat. no. L6115) density centrifugation. Monocytes were separated from lymphocytes by adherence to plastic after a 1 hour incubation in serum-free RPMI-1640 (Gibco) medium. Monocytes were differentiated into macrophages in RPMI-1640 medium supplemented with 100 U/mL penicillin, 100 μg/mL streptomycin and 3% heat-inactivated AB-positive human serum for 7 days. Subsequently, macrophages were cultured in RPMI 1640 medium supplemented with 10% heat-inactivated fetal calf serum (FCS), 100 U/mL penicillin and 100 μg/mL streptomycin.

THP-1 cells were purchased from ATCC (cat. no. TIB-202). THP-1 ACLY knockout cells were described previously (21). Cells were maintained in RPMI-1640 medium supplemented with 10% heat-inactivated FCS, 100 U/mL penicillin, and 100 µg/mL streptomycin.

HEK293T cells, which were purchased from LGC Standards GmbH, were cultured in DMEM medium supplemented with 10% heat-inactivated FCS and 100 U/mL penicillin, and 100 µg/mL streptomycin. Cells were seeded at a density of 1.5 x 10^6^ in 10 cm dishes 24 hours prior to treatment in fresh medium. All cell lines were maintained at 37°C in a humidified atmosphere of 5% CO2.

Cells were treated with indicated concentrations of ND-091143 (Aobius, AOB17806), BMS303141 (Tocris, 4609), 10 μM MK-2206 (Selleckchem, S1078), 10 μM H-89 (Selleckchem, S1582), 50 μM Forskolin (Cayman, Cay11018-1) or 10 ng/mL of LPS (Sigma-Aldrich).

### Generation and cloning of expression vectors

The expression vector was generated by inserting the coding sequence of *homo sapiens* ACLY (Genscript, OHu14076) into the EcoRI linearized pCDH-EF-T2A-MCS-Puro EcoRI vector (System Biosciences, CD527A-1). In brief, the coding sequence of ACLY was amplified by polymerase chain reaction (PCR), elongated with the additional bases of 5’-AGCGAATTCGCCACC-3’ on the 5’ end and 5’-GGATCCTTCGAATTC-3’ on the 3’ end, allowing for subsequent recombination with the vector backbone by In-Fusion^®^ HD cloning (Clontech). Next, the serine to alanine mutant was created by Quickchange site-directed mutagenesis of the TCT into GCT codons using PfuII polymerase (Agilent Technologies). The same mutagenesis approach was employed for the generation of the histidine to alanine (CAT to GCT) mutant of ACLY. This resulted in a phospho-mutant (S455A) and catalytically inactive mutant (H760A) of ACLY, respectively. Plasmids were transformed using heat shock competent *E. coli* bacteria (StellarTM, ST0213, Clontech).

### Transfection and lentiviral transduction

To produce stably transfected cell lines, HEK293T cells were seeded as described above. The next day, HEK293T cells were transfected using the JetPRIME™ transfection reagent (PEQLAB) following the manufacturer’s recommendations. Transfection was carried out using 1.5 µg of the second-generation lentiviral packaging plasmid psPAX2 (Addgene, 12260), 0.5 µg of the envelope expressing plasmid pMD2.G (Addgene, 12259) and 2 µg of the cloned expression vector, encoding for the gene of interest. After 4 hours of incubation, fresh culture medium was added and the cells incubated for another 24 to 48 hours. The supernatant containing infectious lentiviral particles was then collected and centrifuged at 500g for 5 minutes at 4°C and filtered through a 0.2 μm filter. In order to concentrate lentiviral stocks, the Lenti-XTM Concentrator reagent (Takara Bio) was used according to the manufacturer’s instructions. THP-1 cells were seeded into 6-well plates at a density of 1.5x10^6^/well, and transduced with lentiviral particles. Cells were incubated for 24 to 48 hours in culture medium before proceeding to positive selection with puromycin (*In vivo*gen, ant-pr-1) at a concentration of 1 μg/mL for at least 10 days.

### siRNA transfection

Control and ACLY siRNAs (siGENOME human SMARTpool, Horizon Discovery) were transfected into macrophages at a final concentration of 50 nM using HiPerFect transfection reagent (Qiagen) according to the manufacturer’s instructions.

### RNA isolation, reverse transcription and quantitative real-time PCR

Total RNA from macrophages and THP-1 cells was isolated with TRIzol reagent (Life Technologies) according to the manufacturer’s recommendations and quantified using the NanoDrop spectrophotometer (NanoDrop). Reverse transcription of total RNA (1 μg) was carried out using the Maxima first-strand cDNA synthesis kit (ThermoFisher Scientific). Quantitative real-time PCR (Q-PCR) assays were performed with PowerUp SYBR Green Master Mix (Applied Biosystems) using the QuantStudio Real Time PCR System (Applied Biosystems). Primer sequences are available upon request. Target gene expression was calculated using the ΔCt method [rel. expression = 2−(Ct(target)−Ct(reference))] by employing the expression of β2-microglobulin as reference gene.

### RNA sequencing and data analysis

Total RNA from THP-1 cells was isolated using the RNeasy Mini Kit (Qiagen). RNA quality and quantity were checked on an Agilent TapeStation 4150 (Agilent Technologies) and a Qubit 3.0 fluorometer (Thermo Scientific), respectively. RNA integrity numbers for all samples were >9. cDNA libraries for sequencing were prepared from 500 ng of total RNA with Lexogen QuantSeq 3’ mRNA-Seq Library Prep Kit FWD with UDI 12nt set B1 (Lexogen) according to the manufacturer’s instructions. Libraries were quantified on a Qubit and quality was checked on the Agilent TapeStation. Libraries were pooled at the final concentration of 750 nM, spiked with 1% PhiX control library, and sequenced at 9-22 million reads/sample in single-end mode with 75nt read length on the NextSeq 2000 platform (Illumina) with a NextSeq 1000/2000 P2 kit. Demultiplexed FASTQ files were generated with bcl2fastq2 software (Illumina). Processed reads were subsequently mapped to the human genome (GRCh38) using the Bluebee QuantSeq data analyses pipeline (Lexogen). Briefly, reads were quality and adapter trimmed using Bbduk from the bbmap suite before mapping against the human genome GRCh38 using STAR Aligner with modified ENCODE settings. HTSeq-count was used for gene read counting. The counts were utilized to identify differentially expressed genes using DESeq2. Differences in gene expression were considered significant if padj < 0.01. Normalized count data were further analyzed by gene set enrichment analysis (GSEA) using GenePattern online portal (https://www.genepattern.org/). Sequencing data are available on the gene expression omnibus (GEO) platform under accession number GSE216003.

### Histone acid extraction

Cell pellets were lysed in PBS containing 0.5% Triton X-100, 5 mM sodium butyrate and protease inhibitors for 10 minutes. Lysates were subsequently centrifuged for 30 s at 16000g, 4°C. Pellets containing nuclei were incubated with 0.2 M HCl overnight at 4°C. Following a 10 minute centrifugation at 500g at 4°C, supernatants were neutralized with 1 M NaOH, mixed with 1x Laemmli buffer (5x buffer consists of 2% sodium dodecyl sulfate, 62.5 mM Tris-HCl, pH 6.8, 10% glycerol, 50 mM dithiotreitol) and incubated for 5 minutes at 95°C prior to gel electrophoresis.

### Western blotting

Total cell lysates were prepared by scraping cells into lysis buffer (50 mM Tris-HCl, pH 8, 150 mM NaCl, 5 mM EDTA, 10 mM NaF, 1 mM Na_3_VO_4_, 0.5% NP-40), containing 1 mM PMSF and protease inhibitors (Complete, 11697498001, Roche) followed by sonication. Cell lysates were centrifuged at 10000g for 10 minutes at 4°C. The supernatant was collected and heat-denatured with 1x Laemmli buffer for 5 minutes at 95°C.

Protein lysates were resolved on 7.5% - 15% SDS polyacrylamide gels followed by transfer on Protran™ nitrocellulose membranes. Non-specific binding was blocked using 5% milk or 5% BSA in TBST (50 mM Tris/HCl, 140 mM NaCl, pH 7.2, 0.05% Tween-20) for 1 hour at room temperature. The following primary antibodies were utilized by incubating the membranes at 4°C overnight: phospho-ACLY (S455) (#4331), histone H3 acK14 (#7627), histone H3 acK23 (#14932, all Cell Signalling Technology), ACLY (15421-1-AP, Proteintech), histone H3 acK9 (04-1003, Merck Millipore), histone H3 acK27 (C15410174, Diagenode), histone H3 (07-690, Merck Millipore), nucleolin (sc-13057, Santa-Cruz). For protein detection, membranes were incubated with IRDye 680 or IRDye 800-coupled secondary antibodies (LICOR Biosciences) in 5% Milk in TBST for 1 hour at room temperature. Immunological detection was carried out using the Odyssey infrared imaging system (LI-COR Biosciences) and when applicable densitometrically analyzed by employing the Studio™ Lite software (LICOR Biosciences).

### ACLY activity assay

ACLY activity was measured by a coupled enzymatic assay according to Srere (22). THP-1 cells were lysed for 10 minutes on ice with Cell Lysis Buffer (20 mM Tris pH 8.0, 100 mM NaCl, 70 mM KCl, 1 mM MgCl_2,_ 0.2 mM EDTA, 2 mM DTT and 0.5% NP-40). Cell lysates were then centrifuged for 10 minutes at 16000g and the supernatant was then collected. Test buffer (150 mM Tris pH 8.0, 10 mM MgCl_2_, 20 mM sodium citrate, 2 mM dithiotreitol) containing 5 mM ATP, 300 µM CoA, 500 U/ml malate dehydrogenase, 100-200 µM NADH was added to the cell lysate supernatant in a 1:10 dilution, and A_340_ of the samples in the presence or absence of ATP was measured at the beginning and after 15 minutes incubation at 30°C using the BioSpectrometer**^®^** (Eppendorf). Values were normalized to total protein concentration.

### Cytometric bead array

For determination of IL6 and CCL2 levels in THP-1 supernatants Cytometric Bead Array Flex Sets (BD Biosciences) were used according to manufacturer’s instructions. THP-1 cells plated at 0.75x10^6^/ml in 2 ml medium were incubated with 100 ng/ml LPS for 24 hours prior to analysis. Samples were measured using a FACSymphony A5 flow cytometer (BD Biosciences).

### Chromatin immunoprecipitation

THP-1 cells plated at 0.75x10^6^/ml in 6.5 ml medium were treated with 100 ng/ml LPS for 2 hours, fixed in 1% paraformaldehyde, quenched with 0.125M glycine and washed in PBS. Cells were lysed in buffer I (20mM Tris-HCl pH 8.0, 85mM KCl, 0.5% NP-40) to separate the nuclei and the nuclear pellet was lysed in 200µl nuclei lysis buffer (50mM Tris-HCl pH 8.0, 10mM EDTA, 1% SDS) and sonified with Branson Sonifier. Soluble chromatin was diluted with dilution buffer (0.01% SDS, 1.1% Triton X 100, 1.1mM EDTA, 20mM Tris-HCl pH 8.0, 167mM NaCl). The lysate was pre-cleared with sepharose CL-4B beads for 1h and 1% of input was stored at 4°C. The rest of soluble chromatin was incubated overnight at 4°C with 1.5µg of primary antibodies against Lys27-acetylated histone H3 (C15410174, Diagenode) or rabbit IgG (PP64B, Sigma-Aldrich). Protein A/G beads (sc-2003, Santa-Cruz Biotechnology) were added to pull down antibody-protein complexes for 1h at 4°C. The beads were washed once with low salt buffer (0.1% SDS, 1% Triton-X100, 2mM EDTA, 20mM Tris-HCl pH 7.4, 150mM NaCl), once with high salt buffer (0.1% SDS, 1% Triton-X100, 2mM EDTA, 20mM Tris-HCl pH 7.4, 500mM NaCl) and with LiCl buffer (250mM LiCl, 10mM Tris-HCl, pH7.4, 1% NP-40, 1% sodium deoxycholate, 1mM EDTA) at 4°C and twice with TE-buffer at room temperature. The beads were then eluted in 200µl of elution buffer (100mM NaHCO3, 1% SDS) at 55°C. The eluate was reverse crosslinked with RNAse and proteinase K at 65°C for 4h. The decrosslinked DNA was then purified using Qiagen PCR purification kit (Cat. No. 28106) and eluted in 80µl of elution buffer. DNA amounts were quantified using Q-PCR assays with PowerUp SYBR Green Master Mix (Applied Biosystems) and QuantStudio Real Time PCR System (Applied Biosystems).

### Statistical analysis

Data are presented as means ± SEM of at least three independent experiments. Data were analyzed using Student unpaired, two-tailed t test or by one-way analysis of variance (ANOVA) with Bonferroni *post-hoc* means comparison using GraphPad Prism 7.0. Asterisks indicate significant differences between experimental groups (*p<0.05, **p<0.01, ***p<0.005, ns, not significant).

### Ethics

Investigations were conducted in accordance with the ethical standards and following the Declaration of Helsinki as well as national and international guidelines and have been approved by the authors’ institutional review board. The ethics committee of Goethe-University waived the necessity of written informed consent when using the buffy coats from anonymized blood donors.

## Results

### ACLY KO and catalytically inactive H760A mutant cell lines show impaired growth and histone acetylation

**Figure 1:**
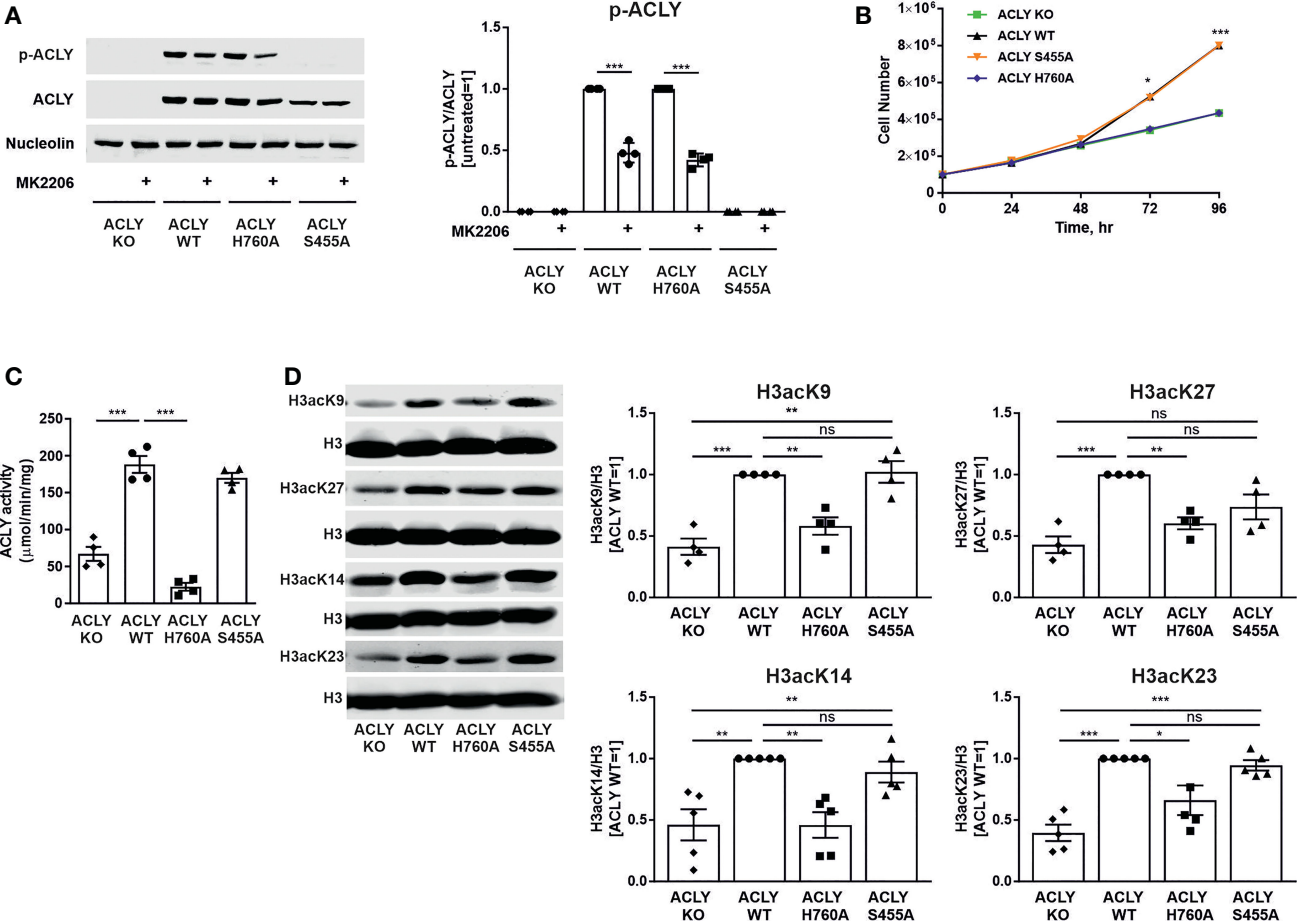
ACLY knockout and catalytically inactive H760A mutant cell lines show impaired growth and reduced basal histone acetylation. **(A)** Western blot analysis of ACLY phosphorylation upon treatment with 10 μM MK2206 for 30 minutes. **(B)** Growth curves. **(C)** ACLY enzymatic activity. **(D)** Basal histone H3 acetylation expression at K9, K27, K14 and K23 in ACLY knockout (KO), WT, H760A and S455A THP-1 cells. Data represent mean values ± SE of 3-5 independent experiments. * p<0.05, ** p<0.01, *** p<0.001, ns, non significant.

In order to test how ACLY phosphorylation at serine 455 influences ACLY function towards metabolic and epigenetic regulation, several THP-1 cell lines were created. Our previous studies showed variability of gene expression between single cell-derived ACLY knockout clones, making a direct comparison with ACLY wild-type cells questionable (21). Therefore, we lentivirally transduced ACLY knockout THP-1 cells with constructs coding for a wild-type (WT) ACLY, catalytically inactive (H760A), or phosphorylation-deficient (S455A) ACLY mutant. **Figure 1A** shows the successful reintroduction of ACLY in the WT, H760A, or S455A mutant cell lines. In contrast to ACLY-deficient and phosphorylation-deficient cell lines, WT and catalytically inactive H760A mutant ACLY retained phosphorylation at serine 455. This phosphorylation appears to be partially driven by Akt, as a 50% reduction in phosphorylation occurred upon treatment with an Akt inhibitor MK2206 (**Figure 1A**). Predictably, and confirming our previous data (21), both ACLY-deficient and H760A THP-1 cell lines showed attenuated growth in comparison to WT and S455A cells (**Figure 1B**). This is likely due to a well-known impairment of *de novo* lipogenesis observed in cell lines with a loss of ACLY activity, which has a direct impact on proliferation (23). Furthermore, ACLY activity was significantly reduced in the ACLY knockout and H760A THP-1 cell lines, but remained intact in S455A cells (**Figure 1C**). Accordingly, the knockout and inactivation of the catalytic activity of ACLY, but not the S455A substitution, in THP-1 cells showed a marked reduction of basal histone H3 acetylation at lysine residues 27, 9, 14 and 23 (**Figure 1D**). Interestingly, nuclear levels of S455 ACLY were elevated as compared to WT or H760A ACLY (**Supplementary Figure 1**).

### ACLY exerts inhibitory effects on pro-inflammatory cytokine expression in response to LPS without affecting cholesterol-handling genes

**Figure 2:**
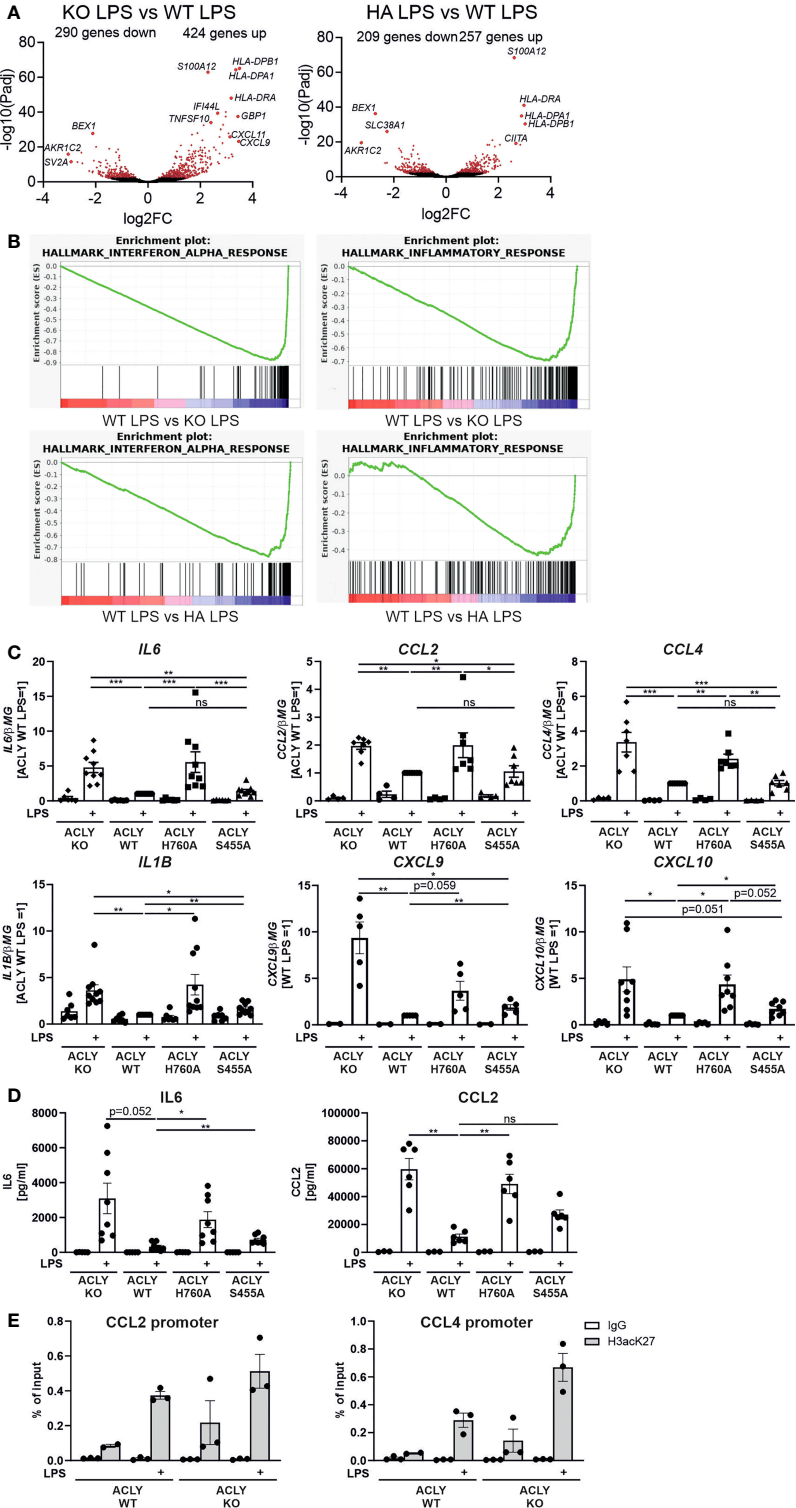
ACLY knockout enhances pro-inflammatory gene expression in THP-1 cells. **(A)** Volcano plot of differentially expressed genes in ACLY knockout vs WT or H760A vs WT THP-1 cells treated with 100 ng/ml LPS for 3 hours. **(B)** GSEA enrichment plots for indicated gene sets comparing LPS-stimulated ACLY knockout vs WT or H760A vs WT THP-1 cells. **(C)** mRNA expression of indicated genes in ACLY knockout, WT, H760A and S455A THP-1 cells treated with 100 ng/ml LPS for 3 hours. **(D)** Secretion of IL6 and CCL2 by WT, H760A and S455A THP-1 cells treated with 100 ng/ml LPS for 24 hours. **(E)** Association of K27-acetylated histone H3 with promoters of CCL2 and CCL4 genes analyzed by ChIP. Data represent mean values ± SE of 3-9 independent experiments. * p<0.05, ** p<0.01, *** p<0.001, ns, non significant.

The role of ACLY in macrophage polarization is still poorly understood, and different experimental settings give differing results (12, 14, 15). We investigated how ACLY WT, knockout, H760A, and S455A THP-1 cells respond to pro-inflammatory stimulation with LPS. Thus, we conducted RNA sequencing analyses in THP-1 cells with or without 3-hour LPS stimulation. These analyses revealed marked differences in gene expression patterns between ACLY WT and ACLY-deficient (KO) THP-1 cells or cells expressing catalytically inactive ACLY H760A (HA), showing 714 and 466 differentially expressed genes under LPS-stimulated conditions, respectively (padj < 0.01) (**Figure 2A**). Most of these changes were already observed in the unstimulated state (**Supplementary Figure 2A**). Gene set enrichment analysis (GSEA) revealed strong enrichment of inflammatory and interferon response genes in LPS-treated ACLY-deficient cells as compared to WT THP-1 cells (**Figure 2B**; **Supplementary Figure 2B**). In contrast, S455A THP-1 cells behaved very similarly to WT cells, showing only 5 up-regulated genes (CXCL11, IFI44, GBP1, EPSTI1, IFIT2) as compared to WT cells after LPS stimulation, while none up-regulated without LPS treatment (padj < 0.01) (**Supplementary Tables S1**, **S2**). Q-PCR analyses confirmed elevated expression of pro-inflammatory response genes in ACLY knockout and H760A cell lines after LPS stimulation with attenuated responses in S455A THP-1 cells (**Figure 2C**). The increased response to LPS persisted when mRNA expression was analyzed 24 hours after stimulation (**Supplementary Figure 3**). Confirming mRNA expression, protein secretion of IL6 and CCL2 into supernatants of LPS-treated ACLY knockout and H760A THP-1 cells was also elevated as compared to WT (**Figure 2D**). Interestingly, in contrast to global decreases of histone H3 K27 acetylation, chromatin immunoprecipitation (ChIP) experiments revealed a tendency to increased association of K27-acetylated histone H3 with promoters of CCL2 and CCL4 (**Figure 2E**) in ACLY knockout THP-1 cells. ACLY knockout murine bone marrow-derived macrophages (BMDMs) showed up-regulation of several genes related to cholesterol synthesis and efflux (14). On the contrary, mRNA expression of cholesterol handling genes such as ABCG1, ABCA1, FASN or DHCR24 was not significantly altered in our cell lines (**Supplementary Figure 4**). We also did not notice differences in cholesterol content between WT, knockout, H760A or S455A cell lines (data not shown). However, acyl-CoA synthetase short chain family member 2 (ACSS2) mRNA was slightly up-regulated in the knockout cell line, suggesting a compensatory mechanism to rescue acetyl-CoA production by the acetate recycling function of this enzyme (23, 24).

### Impact of Akt and PKA inhibition on ACLY phosphorylation, LPS-induced pro-inflammatory cytokine production and histone acetylation in THP-1 cells

**Figure 3:**
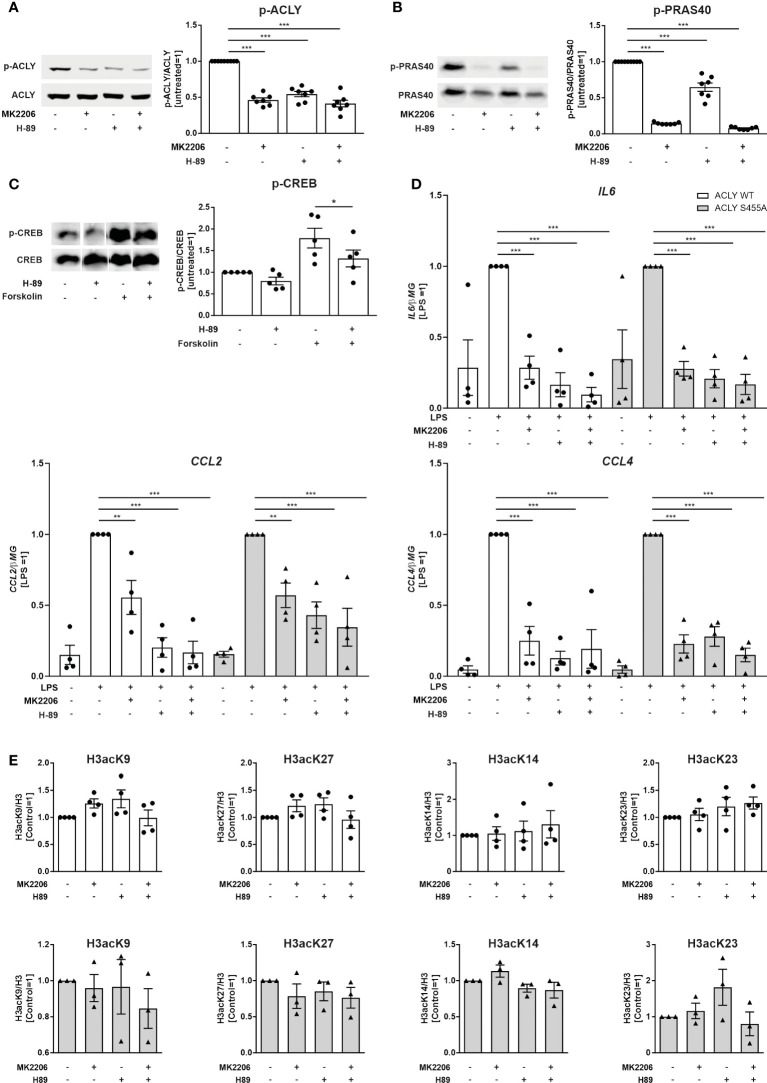
Effects of Akt and PKA inhibition on ACLY phosphorylation, LPS-induced pro-inflammatory cytokine production and basal histone acetylation in THP-1 cells. **(A)** Western blot analysis of ACLY phosphorylation in ACLY WT cells upon treatment with 10 µM MK2206 or 10 µM H-89 for 30 minutes. **(B)** Western blot analysis of PRAS40 phosphorylation in ACLY WT cells upon treatment with 10 µM MK2206 or 10 µM H-89 for 30 minutes. **(C)** Western blot analysis of CREB phosphorylation upon treatment with 10 µM H-89 or 50 µM forskolin for 30 minutes **(D)** mRNA expression of IL6, CCL2 and CCL4 in ACLY WT and S455A THP-1 cells following treatment with 10 µM MK2206 or 10 µM H-89 for 30 minutes and 100 ng/ml LPS for 3 hours. **(E)** Western blot analysis of histone H3 acetylation at K9, K27, K14 and K23 in ACLY WT and S455A cells after treatment with 10 µM MK2206 or 10 µM H-89 for 30 minutes. Data represent mean values ± SE of 3-7 independent experiments. * p<0.05, ** p<0.01, *** p<0.001.

Phosphorylation of ACLY serine 455 was reported to be carried out by Akt and PKA enzymes (17, 18). We found that ACLY serine 455 basal phosphorylation is partially mediated by Akt and PKA in THP-1 cells, since the Akt inhibitor MK2206 and the PKA inhibitor H-89 (**Figure 3A**) diminished it. The proline-rich Akt substrate of 40 kDa (PRAS40) is a specific downstream substrate of Akt (25). Therefore, we used PRAS40 as a marker to confirm that the observed effects on ACLY phosphorylation are a result of Akt action. PRAS40 phosphorylation was significantly reduced after 30 minutes, when treated with 10 μM MK2206. While phospho-PRAS40 is also significantly diminished after the treatment with H-89, it is not as marked (**Figure 3B**). To monitor PKA activity, we assessed phosphorylation levels of the PKA substrate cAMP response element-binding protein (CREB) (26). The PKA inhibitor H-89 reduced CREB phosphorylation in cells treated with the adenylyl cyclase activator forskolin, confirming its ability to inhibit PKA in THP-1 cells (**Figure 3C**). These findings indicate that basal ACLY phosphorylation may be a result of, both Akt and PKA. We next assessed whether inhibition of Akt or PKA influences LPS-induced cytokine production. We observed that both, THP-1 WT and S455A cell lines responded to treatments with MK2206 and H-89, alone or in combination, with decreased expression of IL6, CCL2 and CCL4 after LPS stimulation (**Figure 3D**). These results suggest that MK2206 and H-89 suppress inflammatory responses independently of their effects on ACLY phosphorylation at serine 455. Additionally, we analyzed the effects of Akt or PKA inhibitors on basal histone acetylation in THP-1 cells. Histone H3 acetylation at Lys9, Lys27, Lys14 or Lys23 remained unaffected by Akt/PKA inhibition in both ACLY WT and ACLY S455A cell lines (**Figure 3E**), confirming that activities of Akt and PKA do not influence ACLY function.

### Pharmacological ACLY inhibition or ACLY silencing do not recapitulate the pro-inflammatory phenotype of an ACLY knockout

**Figure 4:**
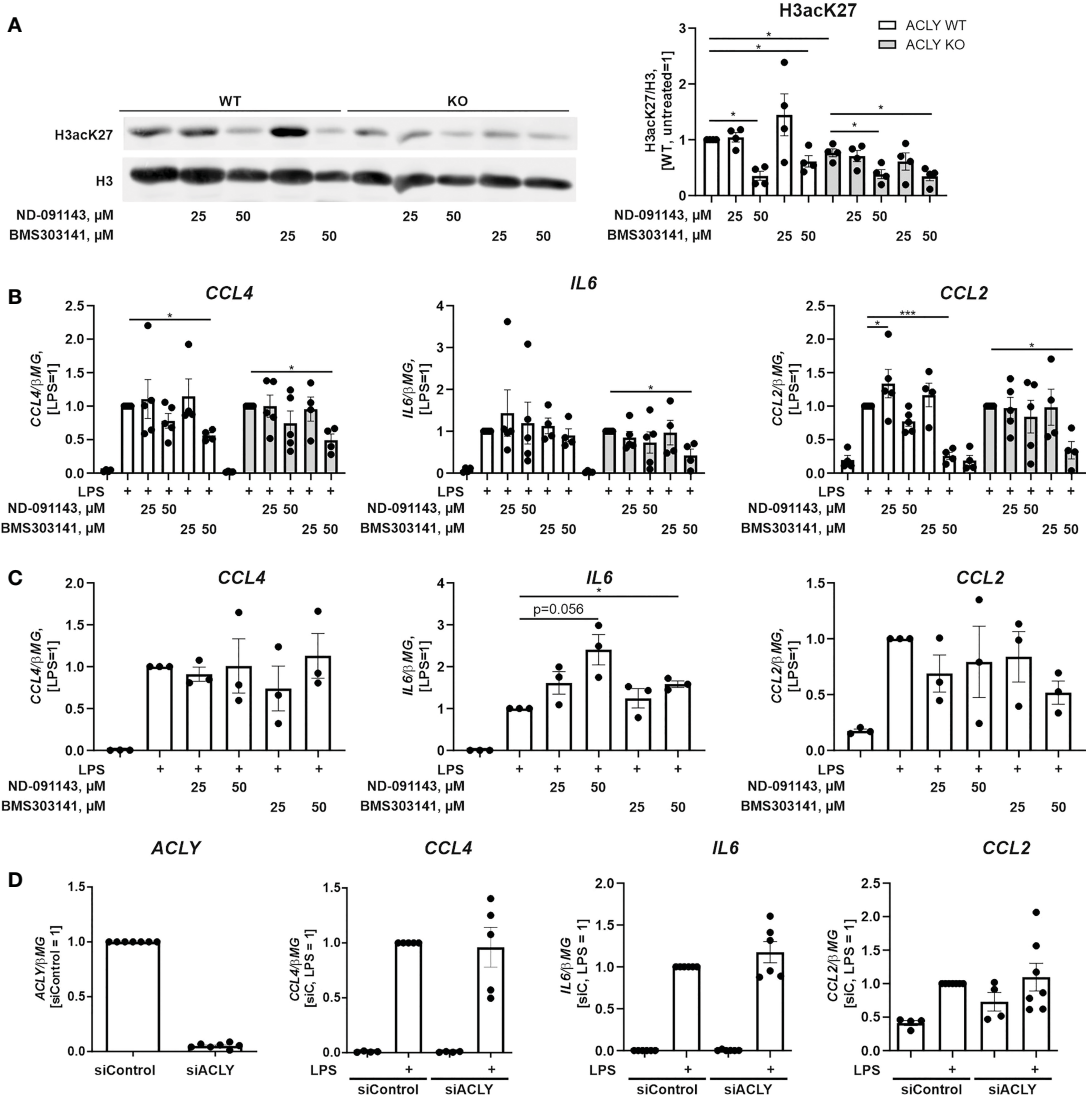
Pharmacological ACLY inhibition or ACLY silencing do not recapitulate pro-inflammatory phenotype of ACLY knockout. **(A)** Western blot analysis of histone H3 acetylation at K27 in ACLY WT and knockout THP-1 cells upon treatments with 25 and 50 μM ND-091143 or BMS303141 for 24 hours. **(B)** mRNA expression of IL6, CCL2 and CCL4 in ACLY WT and knockout THP-1 cells treated with 25 and 50 μM ND-091143 or BMS303141 for 24 hours and 100 ng/ml LPS for 3 hours. **(C)** mRNA expression of IL6, CCL2 and CCL4 in human macrophages treated with 25 and 50 μM ND-091143 or BMS303141 for 24 hours and 100 ng/ml LPS for 3 hours. **(D)** mRNA expression of ACLY, IL6, CCL2 and CCL4 in human macrophages transfected with control (siControl) or ACLY siRNA for 96 hours prior to treatment with 100 ng/ml LPS for 3 hours. Data represent mean values ± SE of 3-4 independent experiments. * p<0.05, *** p<0.001.

Recent studies revealed discrepancies between inflammatory responses of ACLY knockout BMDMs and macrophages where ACLY expression was silenced or pharmacologically inhibited. We explored the response of ACLY WT or knockout THP-1 cell lines towards two pharmacological ACLY inhibitors. BMS303141 was shown to be anti-inflammatory in murine macrophages at 50 µM (12), whereas NDI-091143 is a recently described allosteric ACLY inhibitor (27). Analyzing histone H3 acetylation at Lys27 revealed that at inhibitor concentration of 25 µM no significant alterations were observed after 24 hour-treatments (**Figure 4A**). At 50 µM, both inhibitors reduced histone acetylation. However, this effect was observed both in WT and knockout cell lines, suggesting that at this concentration the reduction of histone acetylation may be at least partly due to off-target effects of the drugs. Next, we exposed ACLY inhibitor-treated cells to LPS and analyzed RNA expression of CCL4, IL6, and CCL2. Only BMS303141 at 50 µM significantly inhibited inflammatory responses of THP-1 cells (**Figure 4B**). However, this effect was also observed in ACLY knockout cells, suggesting that inhibition of inflammatory responses by high BMS303141 concentrations may be unspecific. We also tested BMS303141 and NDI-091143 in LPS-treated human primary macrophages. Here, ACLY inhibitors did not affect the expression of CCL4 and CCL2 (**Figure 4C**). Interestingly, IL6 expression was elevated in cells exposed to LPS after ACLY inhibitor pre-treatment. Analyzing LPS responses in ACLY-silenced cells, which exhibit over 90% reduction of ACLY mRNA, also revealed no significant alterations in the expression of IL6, CCL2, or CCL4 mRNAs (**Figure 4D**) These findings indicate that pharmacological inhibition or silencing of ACLY does not recapitulate the pro-inflammatory phenotype of ACLY knockout cells.

## Discussion

In this study, we questioned how ACLY activity and particularly serine 455 phosphorylation influences histone acetylation and inflammatory gene expression using THP-1 cell lines and human primary macrophages. We obtained evidence that in THP-1 cells, mutation of ACLY at serine 455 did not cause alterations in total histone acetylation as compared to cells expressing WT ACLY. Thus, THP-1 cells expressing WT or S455A ACLY display increased proliferation and show elevated histone acetylation as compared with ACLY-deficient cells or cells expressing catalytically inactive H760A ACLY. Apparently, phosphorylation of serine 455 is not essential for the proper enzymatic function of ACLY in this system. Transcriptome analysis revealed enhanced mRNA expression of pro-inflammatory and interferon response genes in knockout and H760A cells as compared to WT THP-1 cells, while S455A ACLY-expressing cells showed a phenotype almost indistinguishable from the wild type. Altogether, our data suggest that S455A mutation has a very limited influence on ACLY properties causing elevation of inflammatory gene expression. However, increased nuclear location of a S455A mutant ACLY may point to the role of serine phosphorylation in control of nuclear trafficking of this enzyme.

ACLY was one of the first enzymes shown to be phosphorylated by PKA more than 40 years ago (18, 28, 29), with identification of the serine 455 as a target (18). The same site was phosphorylated by an insulin-stimulated kinase (30), later identified as Akt (17). Our results indicated that in unstimulated THP-1 cells, ACLY serine 455 phosphorylation is partly mediated by Akt. This concurs with previous reports, where BMDMs stimulated with either IL-4 (20) or LPS (12) displayed Akt-dependent ACLY phosphorylation at serine 455. At the same time, PKA also contributed to ACLY serine 455 phosphorylation, although to the minor extent. Considering the impact of phosphorylation on ACLY activity, initial studies did not notice differences between the catalytic properties of phosphorylated and dephosphorylated ACLY (29, 31). It was argued that ACLY phosphorylation in response to glucagon was an “incidental occurrence with respect to the program of cAMP-directed regulatory phosphorylation” (30). Later, experiments studying recombinant unphosphorylated ACLY produced in *E.coli* showed a 3-fold increase in ACLY enzymatic activity upon PKA-mediated phosphorylation (16). This increase was blunted in the presence of phosphorylated sugars, such as fructose-1-phosphate, which activated ACLY. It can be argued that endogenous levels of phosphorylated sugars may mask the phosphorylation-dependent difference in ACLY activity. This difference may be revealed by reducing ambient levels of glucose. Indeed, only under conditions of low glucose (1 mM) glioblastoma cells, overexpressing a phosphomimetic S455D ACLY mutant, show higher histone acetylation levels as compared to S455A ACLY-expressing cells. At 10 mM glucose no difference occurred (19). Differences in acetyl-CoA generation were observed in brown preadipocytes overexpressing S455D vs S455A ACLY on the background of Rictor deficiency (32). ACLY serine 455 phosphorylation may also support the enzymatic activity and histone acetylation at specific locations, such as DNA strand breaks (33). Whereas these observations suggest that ACLY phosphorylation may promote acetyl-CoA generation under certain conditions, such as low levels of phospho-sugars, our data do not support the importance of serine 455 phosphorylation under standard growth conditions in our system. Interestingly, conducting ACLY activity assay with lysates of THP-1 ACLY WT and S455A cells, we did not observe differences in activity. This suggests that metabolites or other components of cell lysate may mask the differences in enzymatic activity observed for recombinant unphosphorylated and phosphorylated ACLY (16). We also noted that the S455D ACLY mutant does not exactly mimic ACLY phosphorylation levels, suggested to occur in cells (0.25 mol phosphate/subunit) (16, 34). Thus, data obtained using this mutant should be interpreted with caution.

Importantly, inhibitors of Akt and PKA suppressed inflammatory gene expression in WT as well as in S455A mutant cells, indicating that anti-inflammatory effects of these drugs are independent of ACLY phosphorylation. Furthermore, they did not affect histone acetylation in THP-1 cells, in contrast to suppressive effects of Akt inhibitors in oncogene-transformed mouse pancreatic cells or BMDMs (19, 20). Apparently, metabolic effects of Akt inhibition display considerable heterogeneity between different cell lines.

Our study highlights that THP-1 cells with impaired ACLY activity have a hyperinflammatory gene signature when exposed to LPS. This phenomenon has recently been described in murine BMDMs *in vitro* and *in vivo* (14, 15). Surprisingly, we did not find differences in the expression of cholesterol handling or efflux genes observed in BMDMs, which may be due to interspecies or cell line differences. In contrast to the phenotype of ACLY-deficient THP-1 cells or murine macrophages, studies using pharmacological inhibition or silencing of ACLY showed suppressed responses towards LPS stimulation linked to gene-specific inhibition of histone acetylation (12, 13, 35). Highlighted in a recent review, these discrepancies may reflect long-term adaptations to an ACLY loss as well as off-target effects of the inhibitors (36). Regarding the adaptive mechanisms, increased flux through acetate recycling by the action of ACSS2 can be a potential source of the maintained histone acetylation in ACLY knockout cell lines (23). ACSS2 is an enzyme that contributes to histone acetylation and lipogenesis in cancer cells, especially under stress conditions such as hypoxia or glucose deprivation (24, 37). The loss of ACLY is known to upregulate ACSS2 also in macrophages (14). Similarly, we observed an increased ACSS2 expression in the ACLY-impaired cell lines.

Genetic compensation to a gene knockout is a well described phenomenon showing that different genetic engineering approaches can elicit distinct biological states (38, 39). As such, up-regulation of pro-inflammatory gene expression in cell lines with impaired ACLY activity can be explained by a compensatory mechanism to the loss of enzyme function. Acute pharmacological inhibition or siRNA-mediated knockdown of the enzyme might not give the cells enough time to adapt. The adaptive process how knockout cells rewire other metabolic pathways during a chronic loss of ACLY remains to be investigated. Using small molecule inhibitors of ACLY, while avoiding adaptation, may give rise to off-target effects as we previously described (21). Indeed, our data clearly show that inhibitory effects of BMS303141 on inflammatory response, when used at 50 µM, are independent of ACLY. This emphasizes the general task to carefully titrate inhibitor concentrations in a given cellular system to achieve the optimal on-target action, while minimizing possible off-target effects. Such analyses should apply to ACLY and should help to clarify its role in the epigenetic control of inflammatory responses.

## Limitations of the study

While using THP-1 cells allows genetic manipulations of ACLY, this cancer cell line is clearly distinct from primary human macrophages. Therefore, primary macrophages may have different requirements for ACLY activity and phosphorylation to control inflammatory gene expression. On the other hand, difficulties in transfecting primary macrophages make impossible to compare the impact of different ACLY mutants on metabolism, epigenetics, and gene expression in this system. The mechanistic basis of increased inflammatory gene expression under conditions of ACLY deficiency also requires further investigation. This may also require sampling additional time points after LPS stimulation, as our data hint that ACLY phosphorylation may have a different impact on the inflammatory gene expression at longer stimulation times.

## Data availability statement

The datasets presented in this study can be found in online repositories. The names of the repository/repositories and accession number(s) can be found in the article/**Supplementary Material**.

## Ethics statement

Investigations were conducted in accordance with the ethical standards and following the Declaration of Helsinki as well as national and international guidelines and have been approved by the authors’ institutional review board. The study involving human samples was reviewed and approved by the ethics committee of Goethe University. The ethics committee of Goethe-University waived the necessity of written informed consent when using the buffy coats from anonymized blood donors.

## Author contributions

MD conceived and performed the experiments, analyzed the data, and drafted the manuscript. VT and AM contributed to experimental design. DN and BB participated in study design, data analysis, and writing of the final manuscript draft. All authors contributed to the article and approved the submitted version.

## Funding

This study was supported by the grant from Deutsche Forschungsgemeinschaft (NA429/2-2, SFB1039, Teilprojekt A05).

## Conflict of interest

The authors declare that the research was conducted in the absence of any commercial or financial relationships that could be construed as a potential conflict of interest.

## Publisher’s note

All claims expressed in this article are solely those of the authors and do not necessarily represent those of their affiliated organizations, or those of the publisher, the editors and the reviewers. Any product that may be evaluated in this article, or claim that may be made by its manufacturer, is not guaranteed or endorsed by the publisher.
