# Corrigendum: Impact of ATP-citrate lyase catalytic activity and serine 455 phosphorylation on histone acetylation and inflammatory responses in human monocytic THP-1 cells

**DOI:** 10.3389/fimmu.2024.1434272

**Published:** 2024-07-10

**Authors:** Monica Dominguez, Verena Truemper, Ana Carolina Mota, Bernhard Brüne, Dmitry Namgaladze

**Affiliations:** ^1^ Institute of Biochemistry I, Faculty of Medicine, Goethe-University Frankfurt, Frankfurt, Germany; ^2^ Fraunhofer Institute for Translational Medicine and Pharmacology (ITMP), Frankfurt, Germany; ^3^ German Cancer Consortium (DKTK), Partner Site Frankfurt, Frankfurt, Germany; ^4^ Frankfurt Cancer Institute, Goethe-University Frankfurt, Frankfurt, Germany

**Keywords:** ATP-citrate lyase, histone acetylation, macrophages, metabolism, inflammation

In the published article, there was an error in **Figure 3** as published. The western blot images of pACLY/ACLY (**Figure 3A**) were also mistakenly inserted in **Figure 3B** in place of pPRAS40/PRAS40 images. The corrected **Figure 3** and its caption appear below.

**Figure 3:**
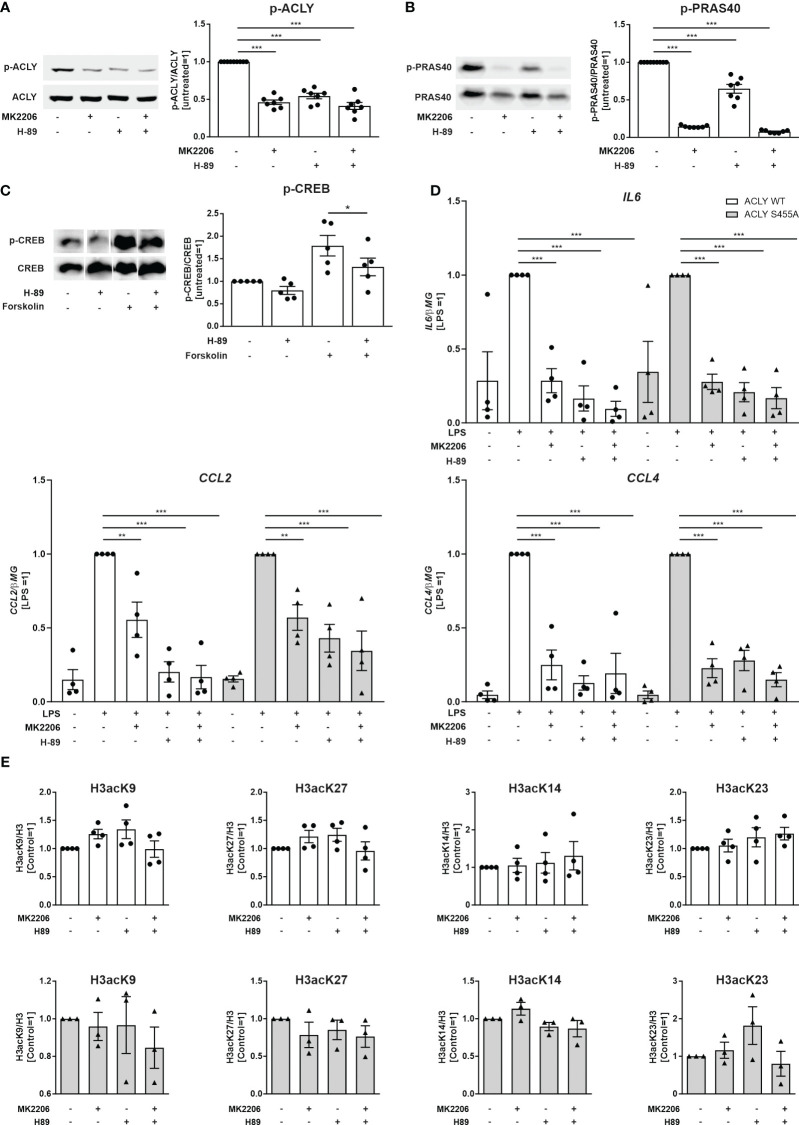
Effects of Akt and PKA inhibition on ACLY phosphorylation, LPS-induced pro-inflammatory cytokine production and basal histone acetylation in THP-1 cells. **(A)** Western blot analysis of ACLY phosphorylation in ACLY WT cells upon treatment with 10 µM MK2206 or 10 µM H-89 for 30 minutes. **(B)** Western blot analysis of PRAS40 phosphorylation in ACLY WT cells upon treatment with 10 µM MK2206 or 10 µM H-89 for 30 minutes. **(C)** Western blot analysis of CREB phosphorylation upon treatment with 10 µM H-89 or 50 µM forskolin for 30 minutes **(D)** mRNA expression of IL6, CCL2 and CCL4 in ACLY WT and S455A THP-1 cells following treatment with 10 µM MK2206 or 10 µM H-89 for 30 minutes and 100 ng/ml LPS for 3 hours. **(E)** Western blot analysis of histone H3 acetylation at K9, K27, K14 and K23 in ACLY WT and S455A cells after treatment with 10 µM MK2206 or 10 µM H-89 for 30 minutes. Data represent mean values ± SE of 3-7 independent experiments. * p<0.05, ** p<0.01, *** p<0.001.

The authors apologize for this error and state that this does not change the scientific conclusions of the article in any way. The original article has been updated.

