# Supplementary figures and images for: Impact of ATP-citrate lyase catalytic activity and serine 455 phosphorylation on histone acetylation and inflammatory responses in human monocytic THP-1 cells
Source: Front Immunol. 2022 Nov 10;13:906127. doi: 10.3389/fimmu.2022.906127 (PMC9686385; doi:10.3389/fimmu.2022.906127)

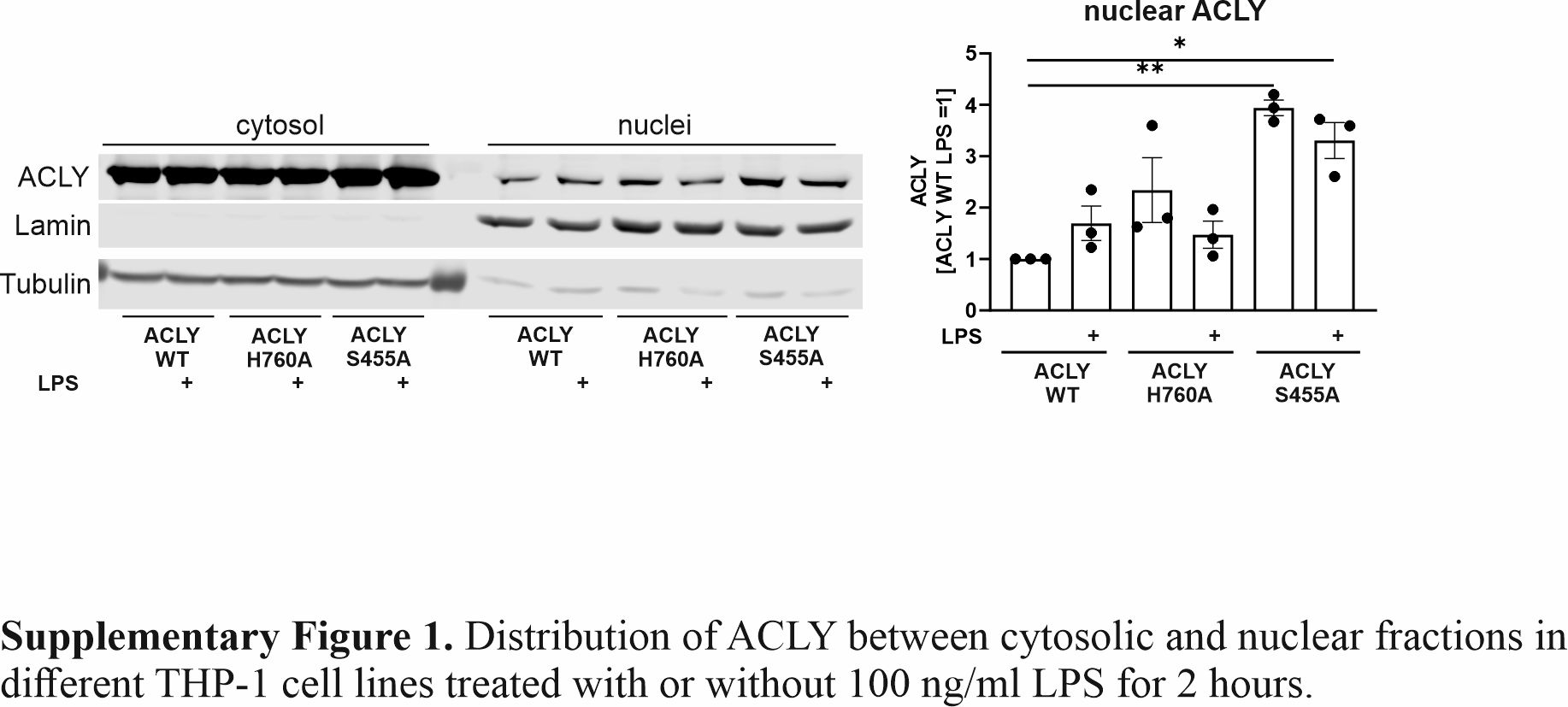

Supplement: Supplementary file 1 [file Image_1.jpeg]

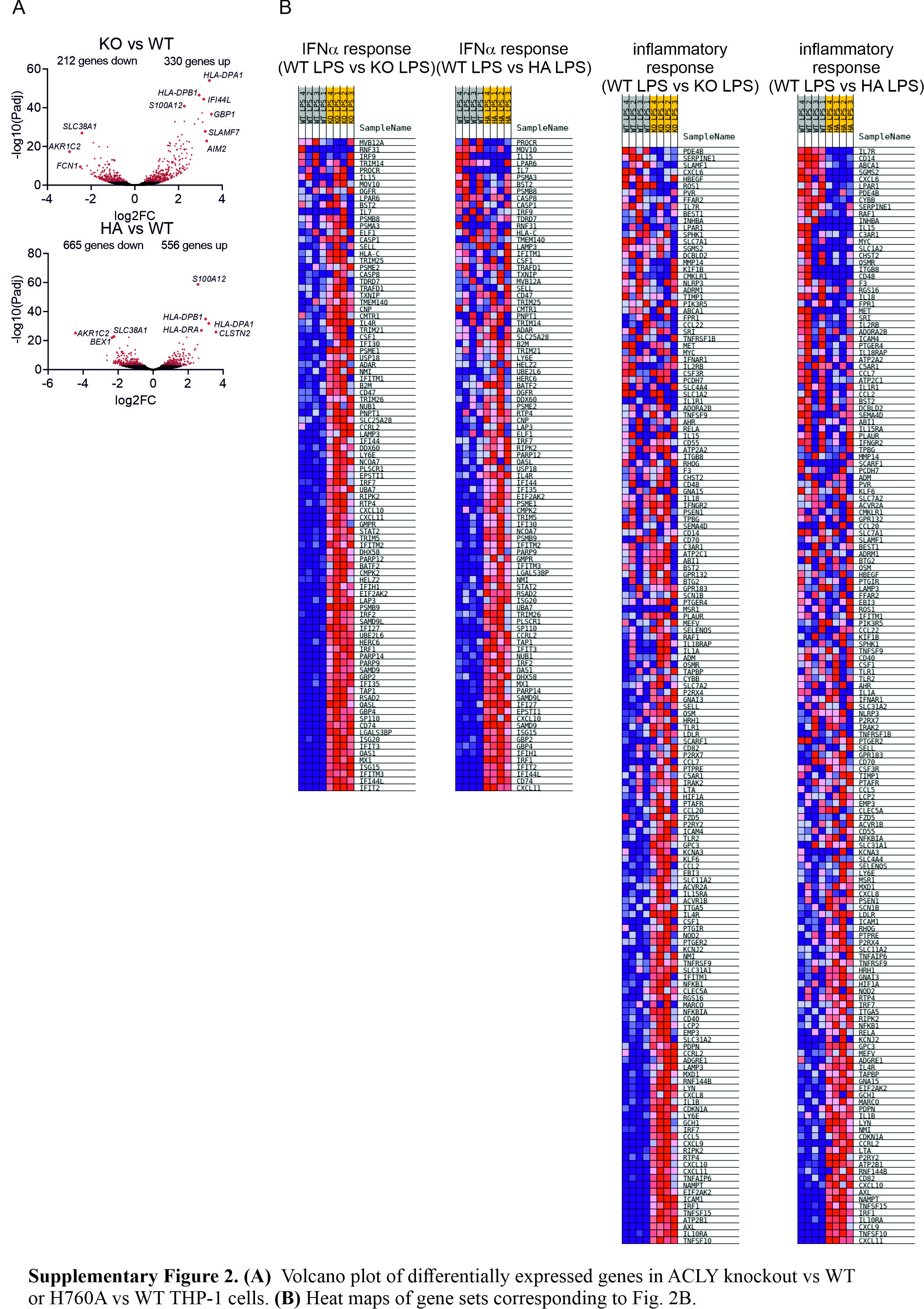

Supplement: Supplementary file 2 [file Image_2.jpeg]

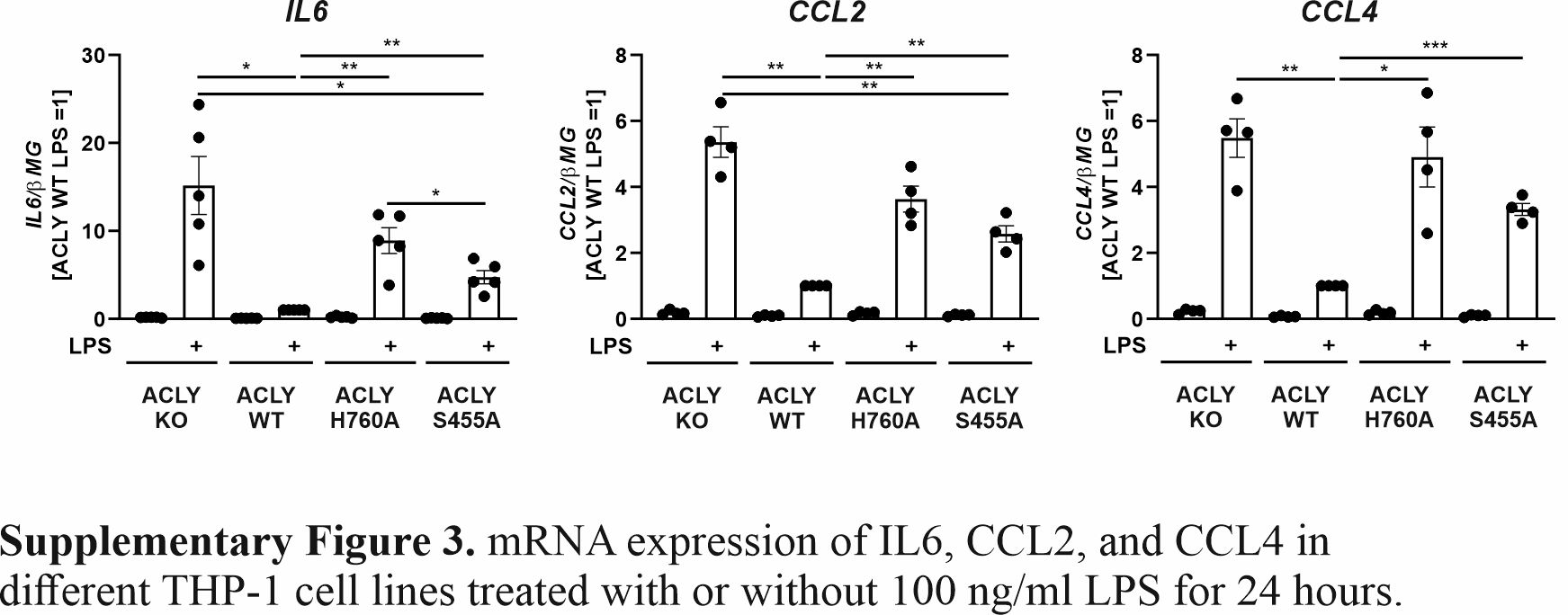

Supplement: Supplementary file 3 [file Image_3.jpeg]

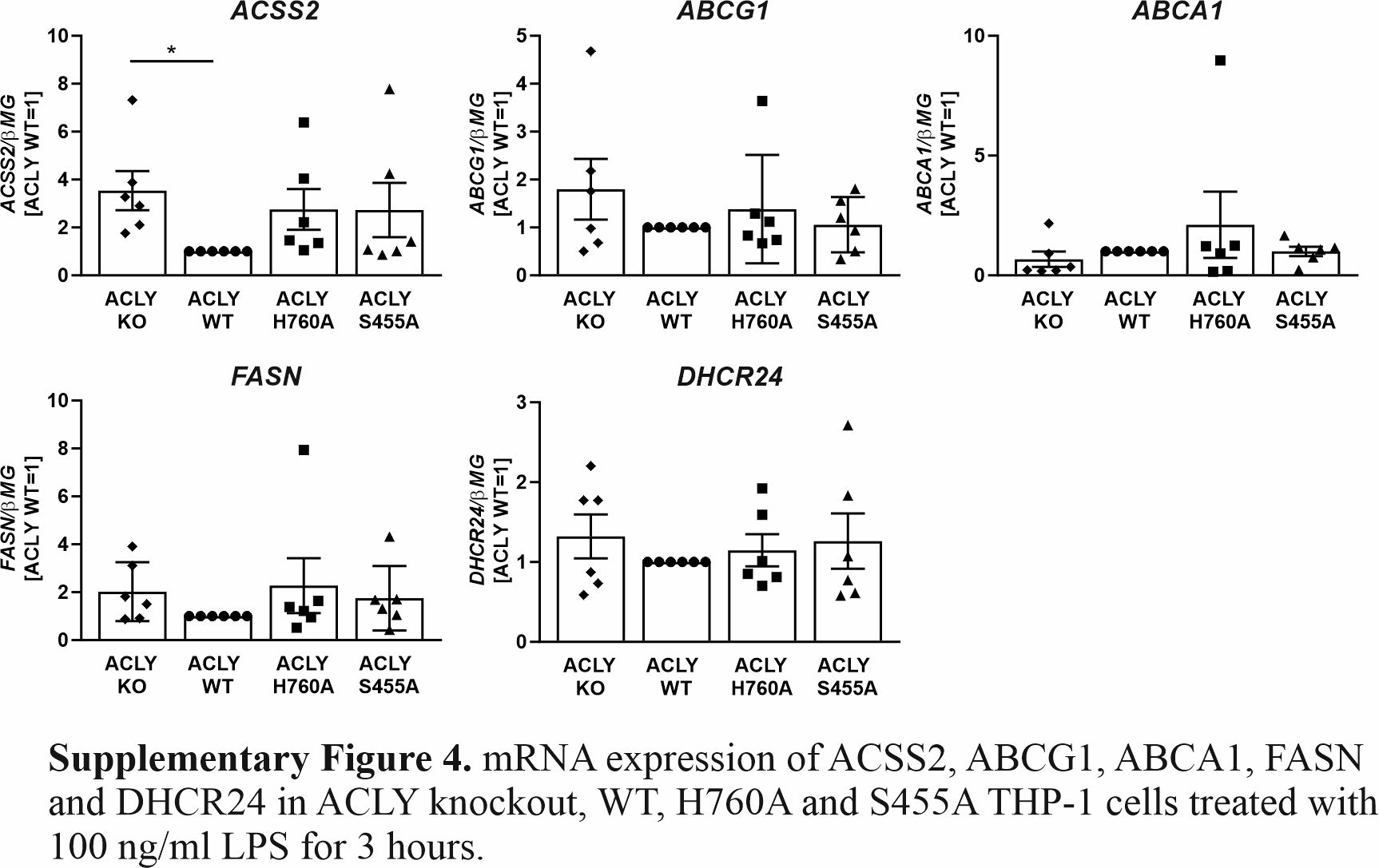

Supplement: Supplementary file 4 [file Image_4.jpeg]
